# Questioning whether IgM Fc receptor (FcµR) is expressed by innate immune cells

**DOI:** 10.1038/s41467-022-29407-0

**Published:** 2022-07-11

**Authors:** Christopher M. Skopnik, René Riedel, Richard K. Addo, Gitta Anne Heinz, Frederik Heinrich, Kazuhito Honjo, Pawel Durek, Philipp Enghard, Mir-Farzin Mashreghi, Andreas Radbruch, Hiromi Kubagawa

**Affiliations:** 1 Deutsches Rheuma-Forschungszentrum in Berlin, 10117 Berlin, Germany; 2 School of Medicine, University of Alabama at Birmingham, Birmingham, AL 35209 USA; 3 Department of Nephrology and Medical Intensive Care, Charité-Universitätmedizin, 10117 Berlin, Germany

**Keywords:** Immunology, Molecular biology, Medical research, Molecular medicine

**arising from** Kubli et al. *Nature Communications* 10.1038/s41467-019-10619-w (2019)

Fc receptors (FcRs) are effector molecules that permit binding to antibodies. Recently, Kubli et al. reported that the FcR for IgM (FcµR) was expressed by non-B cells, i.e., tumor-associated mononuclear phagocytes (TMPs), and was involved in anti-tumor immunity^1^. Here, we have examined whether FcµR is indeed expressed by these cells. Our results do not support their *Fcmr* expression.

Antibody or immunoglobulin (Ig), a key player in humoral immunity, has dual binding activities: to antigens via its amino-terminal variable regions (called Fab) and to effector molecules via its carboxyl-terminal constant region (called Fc). One such effector molecule is a family of cell surface FcRs. FcRs for switched Igs are expressed by many different immune cells, including myeloid cells, and function as central mediators coupling innate and adaptive immune responses. FcµR, identified in 2009, is the newest member of the FcR family. Conflicting views exist especially with regard to its cellular distribution in mice: B cells only *versus* B, myeloid and T cells (see a recent review^2^). Kubli et al. have focused on the function of FcµR in non-B cell populations using his *Fcmr-*deficient (KO) mice. They recently reported that FcµR negatively regulated the anti-tumor activity of TMPs (CD45^**+**^ CD11b^**+**^ MerTk^**+**^ CD64^**+**^) in a melanoma mouse model^1^. *Fcmr* KO mice had increased numbers of TMPs, reduced tumor size and enhanced survival compared to wild type (WT) controls. Single-cell RNA sequence (scRNAseq) analyses of the TMPs from *Fcmr* KO and WT mice revealed a unique TMP subset with enhanced antigen processing/presenting properties in the mutant mice. On the other hand, we and others have focused on FcµR function in B cells^2^, for the simple reason that we have never found expression of FcµR by non-B cells, including myeloid cells, using immunofluorescence analysis with receptor-specific monoclonal antibodies (mAbs) and sensitive reverse transcription polymerase chain reaction assays^3,4^. In this report, we show that *Fcmr* is not expressed by TMPs based on our examination of the scRNAseq data GSE130287 of Kubli et al.^1^ using the R software (version 4.1). We also show our scRNAseq data GSE140133 from splenic IgG memory B cells in C57BL/6 mice^5^ for comparative purposes and provide comments on their data.

As shown in the gene detection histogram based on raw data (Fig. 1a), nearly all of the TMPs (6352 *Fcmr* WT and 8000 *Fcmr* KO cells) had no *Fcmr* transcript reads [i.e., zero unique molecular identifier (UMI)]. Only four *Fcmr* WT cells (0.06%) had 4 (1) or 1 (3) UMIs per cell and six *Fcmr* KO cells (0.08%) had 1 UMI per cell. Notably, the one WT cell with four UMIs also contained transcripts of B cell-specific genes (e.g., *Cd79a*) at similar UMI counts. Our controls included a house-keeping gene (*Gapdh*) and randomly picked, high (*Cd74* and *Cd68*) and low (*Itgam* and *Cd86*) abundance genes. The reads variably ranged: 0 to >300 for *Gapdh*, 0 to >100 for high and 0 to >15 for low abundance genes. To rule out the possibility that *Fcmr* transcripts are somehow undetectable in scRNAseq assessments as is often seen with certain genes, e.g., cytokines, we performed scRNAseq analysis with splenic IgG memory B cells from WT mice (Fig. 1b). Unlike in TMPs, *Fcmr* transcripts were easily detectable in ~75% of the IgG memory B cells at 1 to ~65 UMIs per cell. This is more evident by density curves (Fig. 1c) after normalization on the basis of sequencing depth. One of the high abundance genes *Cd74* in TMPs and *Gapdh* are also highly or clearly expressed by IgG memory B cells. *Cd68* and *Itgam* transcripts were present in TMPs but not in IgG B cells. The expression of *Cd86* was comparable in both cell types. Kubli et al. emphasized *Fcmr* expression by non-B cells in the introduction of the paper, citing several references including theirs, but they did not describe any *Fcmr* transcript results from scRNAseq analysis in the text^1^. Collectively, according to our analysis of their TMP scRNAseq data, we can conclude that there is no evidence for *Fcmr* gene expression by mononuclear phagocytes infiltrating around tumors or TMPs.

**Fig. 1:**
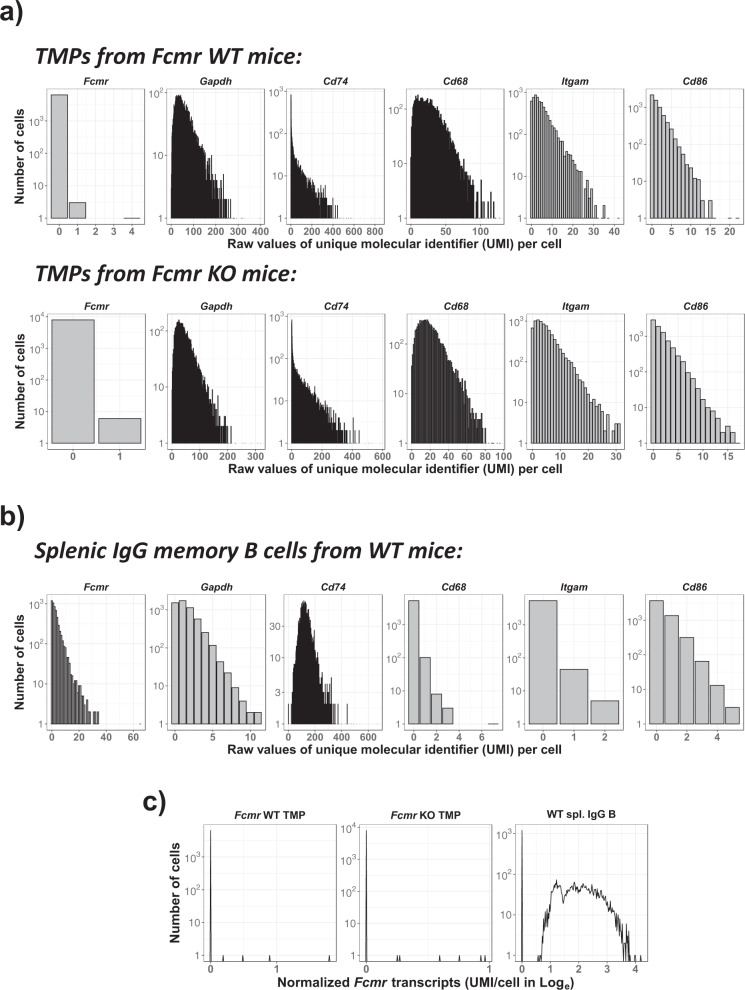
Gene detection histogram. **a**, **b** Raw values for the number of cells and the number of transcript reads (or unique molecular identifier; UMI) of the indicated genes in tumor-associated mononuclear phagocytes (TMPs) from C57BL/6 *Fcmr* WT (upper) and KO (lower) mice (**a**) and IgG memory B cells from C57BL/6 WT spleen (**b**), are plotted on *y*-axes and *x*-axes, respectively. **c** Density curves of normalized UMIs for *Fcmr* transcripts in TMPs from *Fcmr* WT and KO mice and from WT splenic IgG B cells. The TMP data^1^ were derived from GSE130287 and the IgG B cell data^5^ from our scRNAseq analysis GSE140133. Note different scales of the *x*-axis for each gene.

Then, how can we explain the differences observed in *Fcmr-*negative TMP-mediated anti-tumor responses and dendritic cell functions between *Fcmr* KO and WT mice as described in the paper?^1^ Several considerations or potential causes are noteworthy but none of them are definitive. (i) As compared to other *Fcmr* KO mice including ours, the *Fcmr* KO mice of Kubli et al. are unique in extensive targeting of exon 2–8 (~10 kb) and retention of the *Neo* gene in the mouse genome^6,7^. This may account for discrepancies in reported phenotypes among mutant mice^2^. In fact, a difference in granulocyte function between their and our mutant (targeting exon 2–4) mice was noted. Production of reactive oxygen species (ROS) was higher in their *Fcmr* KO granulocytes than WT controls upon stimulation with fMLP in the presence or absence of LPS^7^, but was comparable in our *Fcmr* KO and WT granulocytes (Fig. 2). (ii) Our recent epigenetic analysis of regulatory T (Treg) cells revealed that the 3′ region of *Fcmr* [exon 5 (transmembrane) to exon 8 (cytoplasmic and 3′ untranslated region)] was selectively in an open chromatin configuration when Treg cells were activated, hence the locus may be highly accessible to transcription factors^2,8^. If this selective epigenetic alteration at the 3′ site of *Fcmr* in Treg cells is also the case for TMPs, it may account for differences in TMP cell functions observed in their *Fcmr* KO (missing this 3′ *Fcmr* locus) and WT mice. (iii) Another possibility is suggested by the finding that the conditional deletion of *Fcmr* (exon 4–7) in B cells unexpectedly resulted in impaired proinflammatory T cell responses and immune protection in an acute viral infection^9^. The authors suggested that FcµR on B cells might indirectly regulate T cell functions, although it remains unclear how this would work. Nevertheless, this indirect effect may not be the case for the TMP’s anti-tumor effects, because the above KO TMP-mediated reduction of tumor size and enhanced mouse survival were also observed with their conditional deletion of *Fcmr* (only exon 4) in myeloid, but not in B, cells (see Fig. 1g and Supplementary Fig. 1d, respectively, in the paper^1^). Thus, the molecular basis for functional differences observed in *Fcmr* KO and WT control mice by Kubli et al. is puzzling, but we would emphasize that cell surface FcµR is not directly involved in such a distinction as depicted their model (Fig. 6f)^1^.

**Fig. 2:**
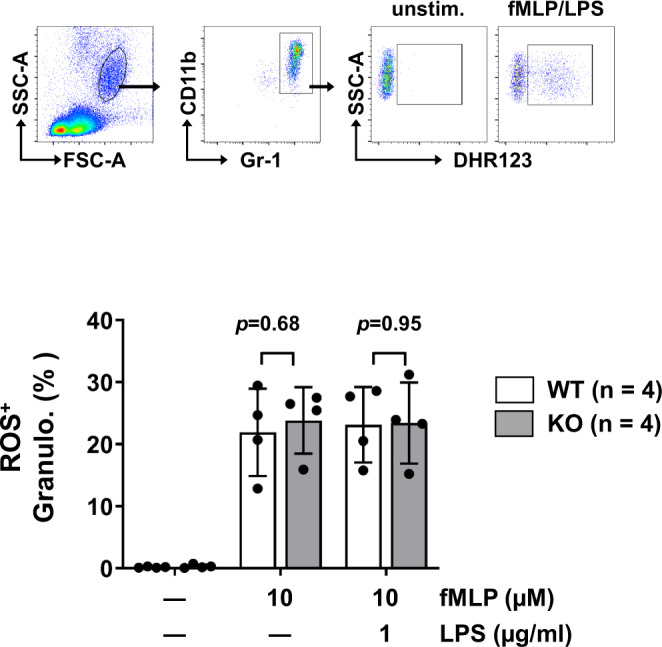
Production of reactive oxygen species (ROS) by granulocytes. Blood granulocytes from our *Fcmr* KO and WT C57BL/6 female mice of 8–12 weeks of age were loaded with 10 µM dihydrorhodamine 123 (DHR123), stimulated with or without 10 µM *N*-formyl-methionine-leucine-phenylalanine (fMLP) in the presence or absence of lipopolysaccharide (LPS; 1 µg/ml), and stained with PE-labeled Gr-1 and PE/Cy7-labeled CD11b mAbs, before lysis of erythrocytes and flow cytometric analysis. Top: Gating strategy for DHR123^**+**^ Gr-1^**+**^ CD11b^**+**^ ROS-producing granulocytes. Bottom: Results are shown in mean ± 1 SD, *n* = 4 *P* values were calculated with an unpaired Student *t-*test and a two-tailed *P* value of <0.05 was statistically significant. Note comparable ROS production between our *Fcmr* KO and WT mice, distinct from enhanced ROS production by their *Fcmr* KO granulocytes^6^.

Given the fact that IgM is the first Ig isotype to appear during phylogeny, ontogeny and immune responses, we also initially thought that FcµR would have a broad cellular distribution thereby serving as a first line of defense against pathogens. Contrary to our prediction, the expression of FcµR is restricted to lymphocytes: B, T, and NK cells in humans and only B cells in mice^2^. Another unexpected finding is the restricted distribution of *FCMR* orthologues to mammals, despite the phylogenetically broad distribution of IgM from jawed vertebrate onward^2,10^. These findings suggest that lymphocyte-specific FcµR must have distinct functions compared to broadly distributed FcRs for switched Ig isotypes. In summary, based on our analysis of the scRNAseq done on TMPs, we demonstrate the absence of *Fcmr* transcripts in such infiltrating monocytoid cells around tumor tissues. Since the authors elegantly described the potential tumor immunity by such TMPs through FcµR without sufficiently backing their *Fcmr* expression results at the single cell level, we would call the readers’ attention to this discrepancy when reading this paper^1^.

## Methods

### Single cell analyses

The scRNAseq data of TMPs (GSE130287) from *Fcmr* WT and KO C57BL/6 mice^1^ and splenic IgG memory B cells (GSE140133) from antigen-immunized WT C57BL/6 mice^5^, which are publicly available (see their repositories below), were reexamined as follows. R package Seurat v4.0.5^11^ was used to read deposited data sets. Raw count matrices were subject to quality control based on: (i) the number of cells where a gene was detected, (ii) the number of genes detected per cell, and (iii) the frequency of UMIs associated with mitochondrial genes. The final filtered matrices contained 16,112 genes and 14,352 cells (6352 *Fcmr* WT and 8000 *Fcmr* KO mice) for TMPs and 13,072 genes and 5436 cells for IgG B cells. Seurats LogNormalize-method with default settings was used to normalize UMI counts.

### Analysis of ROS production

For ROS production, blood was collected from the submandibular vein of *Fcmr* WT and KO C57BL/6 female mice of 8–12 weeks by needle puncture in heparinized microfuge tubes. Twenty microliter of blood were mixed with 180 µl of 10 µM dihydrorhodamine 123 (DHR123; Marker Gene Technologies) in the absence or presence of 10 µM *N-*formyl-methionine-leucine-phenylalanine (fMLP) alone or along with lipopolysaccharide (LPS; Sigma) in DMEM/2% FCS in 96-well flat-bottom plates. After 30 min under 5% CO_2_ at 37 °C, cells were washed with FACS buffer (PBS/2% FCS/0.1% NaN_3_) and stained with PE-labeled GR-1 mAb (clone RB6-8C5) at 0.5 µg/ml and PE/Cy7-labed CD11b mAb (clone M1/70; eBioscience) at 0.67 µg/ml on ice for 20 min, before lysis of erythrocytes with 1 ml of Fix/Lysis buffer (PBS/1% formalin/0.07% saponin) on ice for 10 min. After washing, cells were resuspended in FACS buffer and granulocytes were first gated by high FSC and high SSC characteristics and then by positive for both Gr-1 and CD11b by Accuri C6 Flow Cytometer (BD). DHR123^+^ Gr-1^+^ CD11b^+^ cells were defined as ROS-producing granulocytes. Approximately 50,000–70,000 cell events were acquired and analyzed with FlowJo software (BD). On average 5% of total blood nucleated cells were granulocytes for both groups of mice. Data comparison was performed by unpaired Student *t-*test (GraphPad Prism 9) and a two-tailed *P* value of <0.05 was defined as statistically significant. All studies involving animals were conducted with and after approval of the Landesamt für Gesundheit und Soziales (Lageso) and University of Alabama at Birmingham Institutional Animal Care and Use Committee (IACUC-09195).

### Reporting summary

Further information on research design is available in the Nature Research Reporting Summary linked to this article.

## Supplementary information

Reporting Summary

## Data Availability

The authors declare that all data supporting the findings of this study are available within this article or from the corresponding author upon request. Single cell RNA-sequencing data of TMPs and IgG memory B cells have been deposited in the Gene Expression Omnibus (GEO) repository by the original authors, Kubli et al. and Riedel et al. Accession codes are GSE130287 and GSE140133, respectively.

## Code availability

The R code to reproduce Fig. 1 is deposited at the public GitHub repository https://github.com/Close-your-eyes/NCOMMS-19-25487A.

## References

[1] Kubli SP (2019). Fcmr regulates mononuclear phagocyte control of anti-tumor immunity. Nat. Commun..

[2] Kubagawa H (2019). Functional roles of the IgM Fc receptor in the immune system. Front. Immunol..

[3] Honjo K, Kubagawa Y, Kubagawa H (2013). Is Toso/IgM Fc receptor (FcμR) expressed by innate immune cells?. Proc. Natl Acad. Sci. USA.

[4] Lang KS (2013). Reply to Honjo et al.: Functional relevant expression of Toso on granulocytes. Proc. Natl Acad. Sci. USA.

[5] Riedel R (2020). Discrete populations of isotype-switched memory B lymphocytes are maintained in murine spleen and bone marrow. Nat. Commun..

[6] Choi SC (2013). Mouse IgM Fc receptor, FCMR, promotes B cell development and modulates antigen-driven immune responses. J. Immunol..

[7] Lang KS (2013). Involvement of Toso in activation of monocytes, macrophages, and granulocytes. Proc. Natl Acad. Sci. USA.

[8] Kitagawa Y (2017). Guidance of regulatory T cell development by Satb1-dependent super-enhancer establishment. Nat. Immunol..

[9] Yu J (2018). Surface receptor Toso controls B cell-mediated regulation of T cell immunity. J. Clin. Invest..

[10] Akula S, Mohammadamin S, Hellman L (2014). Fc receptors for immunoglobulins and their appearance during vertebrate evolution. PLoS ONE.

[11] Hao Y (2021). Integrated analysis of multimodal single-cell data. Cell.

