# Supplementary material for: Questioning whether IgM Fc receptor (FcµR) is expressed by innate immune cells
Source: Nat Commun. 2022 Jul 11;13:3951. doi: 10.1038/s41467-022-29407-0 (PMC9273587; doi:10.1038/s41467-022-29407-0)
Supplement: Supplementary file 1 — Reporting Summary [file 41467_2022_29407_MOESM1_ESM.pdf]

nature portfolio

Corresponding author(s): *Hiromi Kubagawa*

Last updated by author(s): *2021/12/17*

Reporting Summary

Nature Portfolio wishes to improve the reproducibility of the work that we publish. This form provides structure for consistency and transparency in reporting. For further information on Nature Portfolio policies, see our Editorial Policies and the Editorial Policy Checklist.

Statistics

For all statistical analyses, confirm that the following items are present in the figure legend, table legend, main text, or Methods section.

n/a	Confirmed	
☐	☒	The exact sample size (*n*) for each experimental group/condition, given as a discrete number and unit of measurement
☐	☒	A statement on whether measurements were taken from distinct samples or whether the same sample was measured repeatedly
☐	☒	The statistical test(s) used AND whether they are one- or two-sided<br>*Only common tests should be described solely by name; describe more complex techniques in the Methods section.*
☒	☐	A description of all covariates tested
☐	☒	A description of any assumptions or corrections, such as tests of normality and adjustment for multiple comparisons
☐	☒	A full description of the statistical parameters including central tendency (e.g. means) or other basic estimates (e.g. regression coefficient) AND variation (e.g. standard deviation) or associated estimates of uncertainty (e.g. confidence intervals)
☐	☒	For null hypothesis testing, the test statistic (e.g. *F*, *t*, *r*) with confidence intervals, effect sizes, degrees of freedom and *P* value noted<br>*Give P values as exact values whenever suitable.*
☒	☐	For Bayesian analysis, information on the choice of priors and Markov chain Monte Carlo settings
☒	☐	For hierarchical and complex designs, identification of the appropriate level for tests and full reporting of outcomes
☒	☐	Estimates of effect sizes (e.g. Cohen's *d*, Pearson's *r*), indicating how they were calculated

Our web collection on statistics for biologists contains articles on many of the points above.

Software and code

Policy information about availability of computer code

Data collection	*FlowJo (version 9&10)*
Data analysis	*R (version 4.1)*

For manuscripts utilizing custom algorithms or software that are central to the research but not yet described in published literature, software must be made available to editors and reviewers. We strongly encourage code deposition in a community repository (e.g. GitHub). See the Nature Portfolio guidelines for submitting code & software for further information.

Data

Policy information about availability of data

All manuscripts must include a data availability statement. This statement should provide the following information, where applicable:

- Accession codes, unique identifiers, or web links for publicly available datasets
- A description of any restrictions on data availability
- For clinical datasets or third party data, please ensure that the statement adheres to our policy

The authors declare that all data supporting the findings of this study are available within this article or from the corresponding author upon reasonable request. Single cell RNA-sequencing data of TMPs and IgG memory B cells have been deposited in the Gene Expression Omnibus (GEO) repository by the original authors, Kubli et al. and Riedel et al. Accession codes are GSE130287 [https://www.ncbi.nlm.nih.gov/geo/query/acc.cgi?acc=GSE130287] and GSE140133 [https://www.ncbi.nlm.nih.gov/geo/query/acc.cgi?acc=GSE140133], respectively.

Field-specific reporting

Please select the one below that is the best fit for your research. If you are not sure, read the appropriate sections before making your selection.

☒ Life sciences ☐ Behavioural & social sciences ☐ Ecological, evolutionary & environmental sciences

For a reference copy of the document with all sections, see nature.com/documents/nr-reporting-summary-flat.pdf

Life sciences study design

All studies must disclose on these points even when the disclosure is negative.

Sample size	*Sample size was determined on the statistical basis of minimal expected effect size.*
Data exclusions	*For reanalysis of scRNAseq data, low-quality single cell transcriptomes were removed in an initial quality control.*
Replication	*Not relevant.*
Randomization	*Randomization is not relevant to the present study.*
Blinding	*Blinding was not performed.*

Behavioural & social sciences study design

All studies must disclose on these points even when the disclosure is negative.

Study description	*N/A*
Research sample	*N/A*
Sampling strategy	*N/A*
Data collection	*N/A*
Timing	*N/A*
Data exclusions	*N/A*
Non-participation	*N/A*
Randomization	*N/A*

Ecological, evolutionary & environmental sciences study design

All studies must disclose on these points even when the disclosure is negative.

Study description	*N/A*
Research sample	*N/A*
Sampling strategy	*N/A*
Data collection	*N/A*
Timing and spatial scale	*N/A*
Data exclusions	*N/A*
Reproducibility	*N/A*
Randomization	*N/A*
Blinding	*N/A*

Did the study involve field work? ☐ Yes ☒ No

Field work, collection and transport

Field conditions	*N/A*
Location	*N/A*
Access & import/export	*N/A*
Disturbance	*N/A*

Reporting for specific materials, systems and methods

We require information from authors about some types of materials, experimental systems and methods used in many studies. Here, indicate whether each material, system or method listed is relevant to your study. If you are not sure if a list item applies to your research, read the appropriate section before selecting a response.

Materials & experimental systems

n/a	Involved in the study	
☐	☒	Antibodies
☒	☐	Eukaryotic cell lines
☒	☐	Palaeontology and archaeology
☐	☒	Animals and other organisms
☒	☐	Human research participants
☒	☐	Clinical data
☒	☐	Dual use research of concern

Methods

n/a	Involved in the study	
☒	☐	ChIP-seq
☐	☒	Flow cytometry
☒	☐	MRI-based neuroimaging

Antibodies

Antibodies used	*For Fig. 2, PE-labeled Gr-1 mAb (eBioscience, Cat No. 12-5931-83, 1:400 dilution, working concentration of 0.5 µg/ml); PE/Cy7-labeled CD11b mAb (eBioscience, Cat no. 25-0112-81, 1:300 dilution, working concentration of 0.67 µg/ml).*
Validation	*Gr-1 mAb: Fleming TJ, et al. J. Immunol. 151:2399, 1993; CD11b mAb: Springer T, et al. Eur. J. Immunol. 8:539, 1978.*

Eukaryotic cell lines

Policy information about cell lines

Cell line source(s)	*N/A*
Authentication	*N/A*
Mycoplasma contamination	*N/A*
Commonly misidentified lines (See ICLAC register)	*N/A*

Palaeontology and Archaeology

Specimen provenance	*N/A*
Specimen deposition	*N/A*
Dating methods	*N/A*

☐ Tick this box to confirm that the raw and calibrated dates are available in the paper or in Supplementary Information.

Ethics oversight	*N/A*

Note that full information on the approval of the study protocol must also be provided in the manuscript.

Animals and other organisms

Policy information about studies involving animals; ARRIVE guidelines recommended for reporting animal research

Laboratory animals	*For Fig. 1, C57BL/6 mice were housed under specific pathgen-free conditions with 12 light/dark cycle at 18-21°C with food and water ad libitum as described in Nat. Commun. 11:2570, 2020. For Fig. 2, Fcmr(-/-) homozygous and Fcmr(+/+) littermate C57BL/6 female mice of 8-12 wk of age were used.(Honjo, K et al. Proc. Natl. Acad. Sci. 109:15882, 2012).*
Wild animals	*No wild mice were used in the present study.*
Field-collected samples	*No field collected samples were used in the present study.*
Ethics oversight	*All studies involving animals were conducted in accordance with and after approval of the Landesamt für Gesundheit und Soziales (Lageso) for Fig. 1 and the University of Alabama at Birmingham (UAB) Institutional Animal Care and Use Committee (IACUC) IACUC-09195 for Fig. 2.*

Note that full information on the approval of the study protocol must also be provided in the manuscript.

Human research participants

Policy information about studies involving human research participants

Population characteristics	*N/A*
Recruitment	*N/A*
Ethics oversight	*N/A*

Note that full information on the approval of the study protocol must also be provided in the manuscript.

Clinical data

Policy information about clinical studies

All manuscripts should comply with the ICMJE guidelines for publication of clinical research and a completed CONSORT checklist must be included with all submissions.

Clinical trial registration	*N/A*
Study protocol	*N/A*
Data collection	*N/A*
Outcomes	*N/A*

Dual use research of concern

Policy information about dual use research of concern

Hazards

Could the accidental, deliberate or reckless misuse of agents or technologies generated in the work, or the application of information presented in the manuscript, pose a threat to:

No	Yes	
☒	☐	Public health
☒	☐	National security
☒	☐	Crops and/or livestock
☒	☐	Ecosystems
☒	☐	Any other significant area

Experiments of concern

Does the work involve any of these experiments of concern:

No	Yes	
☒	☐	Demonstrate how to render a vaccine ineffective
☒	☐	Confer resistance to therapeutically useful antibiotics or antiviral agents
☒	☐	Enhance the virulence of a pathogen or render a nonpathogen virulent
☒	☐	Increase transmissibility of a pathogen
☒	☐	Alter the host range of a pathogen
☒	☐	Enable evasion of diagnostic/detection modalities
☒	☐	Enable the weaponization of a biological agent or toxin
☒	☐	Any other potentially harmful combination of experiments and agents

ChIP-seq

Data deposition

☐ Confirm that both raw and final processed data have been deposited in a public database such as GEO.

☐ Confirm that you have deposited or provided access to graph files (e.g. BED files) for the called peaks.

Data access links<br>*May remain private before publication.*	*N/A*
Files in database submission	*N/A*
Genome browser session<br>(e.g. UCSC)	*N/A*

Methodology

Replicates	*N/A*
Sequencing depth	*N/A*
Antibodies	*N/A*
Peak calling parameters	*N/A*
Data quality	*N/A*
Software	*N/A*

Flow Cytometry

Plots

Confirm that:

☒ The axis labels state the marker and fluorochrome used (e.g. CD4-FITC).

☒ The axis scales are clearly visible. Include numbers along axes only for bottom left plot of group (a 'group' is an analysis of identical markers).

☒ All plots are contour plots with outliers or pseudocolor plots.

☒ A numerical value for number of cells or percentage (with statistics) is provided.

Methodology

Sample preparation	*For splenic IgG B cells in Fig. 1c, please see the original paper of Riedel, et al. in Nat. Commun. 11:2570, 2020. For Fig. 2, blood was collected from the submandibular vein of mice by needle puncture in a heparinized 1.5 ml tube. Twenty µl of blood were mixed with 180 µl of 10 µM Dihydrorhodamine 123 (Marker Gene Technologies) in the absence or presence of 10 µM fMLP or of 10 µM fMLP plus LPS (1 µg/ml; Sigma) in 2% FCS/DMEM in wells of flat bottom 96-well plates. After incubation for 30 min in carbon dioxide incubator, cells were harvested, washed in FACS buffer (2% FCS/PBS/0.1% sodium azide), and stained with PE-Gr-1 mAb (clone RB6-8C5; eBioscience) and PE/Cy7-CD11b mAb (clone M1/70; eBioscience) on ice for 20 min, followed by incubation with Fix/Lysis buffer (1% formaldehyde/0.07% saponin/PBS) on ice for 10 min to lyse erythrocytes. After washing, 50,000-70,000 stained cells were acquired on Accuri C6 Flow Cytometer (BD) and analyzed with FlowJo software (Tree Star).*

Granulocytes were gated on their characteristics of high FSC/high SSC and CD11b(+)/Gr-1(+). On average, 5% of total nucleated cells in blood were granulocytes for both Fcmr KO and WT mice.

Instrument: *For Fig. 1c, please see the original paper of Riedel, et al. in Nat. Commun. 11:2570, 2020. For Fig. 2, Accuri C6 Flow Cytometer (BD).*

Software: *For Fig. 1c, please see the original paper of Riedel, et al. in Nat. Commun. 11:2570, 2020. For Fig. 2, FlowJo (Tree Star; BD).*

Cell population abundance: *For Fig. 1c, please see the original paper of Riedel, et al. in Nat. Commun. 11:2570, 2020. For Fig. 2, approximately 5% of total nucleated cells in blood were granulocytes.*

Gating strategy: *For Fig. 1c, please see the original paper of Riedel, et al. in Nat. Commun. 11:2570, 2020. For Fig. 2, granulocytes were gated on the basis of high FSC and high SSC characteristics and the CD11b(+) and Gr-1(+) phenotype.*

☒ Tick this box to confirm that a figure exemplifying the gating strategy is provided in the Supplementary Information.

Magnetic resonance imaging

Experimental design

Design type: *N/A*

Design specifications: *N/A*

Behavioral performance measures: *N/A*

Acquisition

Imaging type(s): *N/A*

Field strength: *N/A*

Sequence & imaging parameters: *N/A*

Area of acquisition: *N/A*

Diffusion MRI ☐ Used ☐ Not used

Preprocessing

Preprocessing software: *N/A*

Normalization: *N/A*

Normalization template: *N/A*

Noise and artifact removal: *N/A*

Volume censoring: *N/A*

Statistical modeling & inference

Model type and settings: *N/A*

Effect(s) tested: *N/A*

Specify type of analysis: ☐ Whole brain ☐ ROI-based ☐ Both

Statistic type for inference (See Eklund et al. 2016): *N/A*

Correction: *N/A*

Models & analysis

n/a	Involved in the study
☒	☐ Functional and/or effective connectivity
☒	☐ Graph analysis
☒	☐ Multivariate modeling or predictive analysis

Functional and/or effective connectivity	*N/A*
Graph analysis	*N/A*
Multivariate modeling and predictive analysis	*N/A*
